# Airport Spatial Usability in Measuring the Spherical Antenna Properties on Small Aircraft

**DOI:** 10.3390/s21237920

**Published:** 2021-11-27

**Authors:** Ján Labun, Pavol Kurdel, Marek Češkovič, Alexey Nekrasov, Mária Gamcová, Natália Gecejová

**Affiliations:** 1 Faculty of Electrical Engineering and Informatics, Technical University of Košice, Letná 9, 04200 Košice, Slovakia; jan.labun@tuke.sk (J.L.); maria.gamcova@tuke.sk (M.G.); 2 Faculty of Aeronautics, Technical University of Košice, Rampová 7, 04121 Košice, Slovakia; marek.ceskovic@tuke.sk (M.Č.); natalia.gecejova@tuke.sk (N.G.); 3 Institute for Computer Technologies and Information Security, Southern Federal University, Chekhova 2, 347922 Taganrog, Russia; alexei-nekrassov@mail.ru; 4 Department of Radio Engineering Systems, Saint Petersburg Electrotechnical University, Professora Popova 5, 197376 Saint Petersburg, Russia

**Keywords:** emergency locator transmitter system, flight safety, small aircraft, spherical antenna characteristics

## Abstract

The strict safety requirements of air transport for nonstandard placement of electronic onboard systems require an innovative approach to the experimental verification of the placement of these devices. Particular attention is required to the location of these electronic devices’ antenna systems on the fuselage. A prerequisite for determining the location of the antenna and verifying its radiation is a thorough knowledge of the radio communication transmission of onboard electronic systems in cooperation with terrestrial or satellite systems. From this point of view, this article focuses on an innovative method of verifying the spherical radiation characteristics of the antenna of an onboard rescue system emergency locator transmitter (ELT) to assess its communication link with the Cospas-Sarsat satellite system. The measurement is performed on a small sports two-seater aircraft with an antenna placed in an unusual place in the aircraft’s cabin, between the seats. It was impossible to use a suitable nonreflective attenuation chamber for the measurement, so we present a method and procedure for this type of measurement in the open space of an airport. The achieved results prove the plausibility and reproducibility of the measurement. Furthermore, combining several polar radiation characteristics makes it possible to obtain an idea, even if only a part, of the spatial (spherical) radiation characteristic. This article presents a simple method of measuring the characteristics of aircraft antennas when it is not possible to use a suitable professional nonreflective attenuation chamber for measurements for various reasons. This method can also be used on other larger means of transport or other objects that experience the same problem.

## 1. Introduction

Air traffic in today’s modern Europe is primarily focused on flights by ultralight and light aircraft. Dangerous situations are common for this type of air traffic. Due to these situations, the implementation of a collision-avoidance system and a rescue system is desirable, even required. The affordability of this type of aircraft has caused their quantitative increase as a proportion of air traffic, also supported by the increase in flight hours, especially for their use in sports or private passenger transport. The tendency in the future will be to automate the control of this type of aircraft. So, this type of transport will expand even further. For this reason, even stricter requirements will be placed on air safety control [1,2].

In the case of an emergency, all available systems are used to save the life of the pilot and crew [1,2,3,4]. These can be activated in different phases of flight: automatically in the event of an aircraft collision or manually during a flight. In the event of an accident, the ELT emergency location system is automatically started. The ELT system sends an emergency signal when a crash is detected. It thus provides the possibility of rescuing the pilot and crew of the aircraft. Its location on an aircraft is therefore strategically crucial. Equally crucial is the ability to remove the ELT from the aircraft cabin and call for help from a safe distance from the accident site. It is a unique system that is integrated into the air search and rescue service. Thanks to its satellite connection, it allows rescuing persons in almost all countries of the world [3,4,5,6].

This article discusses an ELT that transmits a standard tone on frequencies of 121.5 and 406 MHz when activated. To ensure maximum efficiency of rescue, normative conditions have been established for the use of the ELT system. The use of a functional ELT system is compulsory for aircraft flights from the departure airport to a radius of more than 50 NM (92.6 km). If the ELT is permanently installed onboard, it should be in automatic operation mode before flight. If a portable ELT is available, it must be stored in an easily accessible place and put into automatic operation. The exceptions are flights within 50 NM (92.6 km) of the point of departure and agricultural flights [3,4].

**Figure 1:**
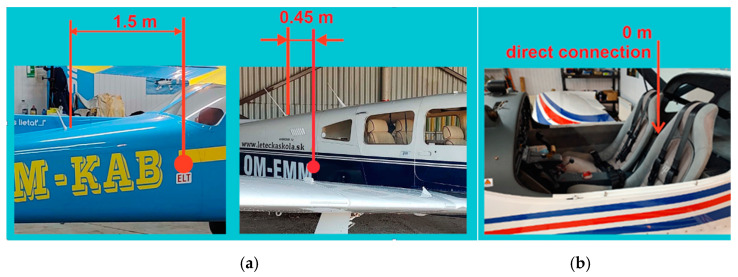
Location of the ELT locating device in the assembly: block–antenna: (**a**) standard location (distance) of antenna and ELT block; (**b**) nontraditional location of antenna and ELT block.

The ELT block is installed/located in an area with the minimal statistical possibility of destruction by airplane crash or fire. Some examples of the location of the ELT block and its antenna on small aircraft are shown in Figure 1. A possible emergency requires determination and a quick sequence of steps leading to rescue. Therefore, the location of the antenna elements of the ELT system must be sophisticated and set according to the aircraft’s design and aerodynamic solutions. In combination with the transmitting antenna, the ELT system must achieve maximum transmitting power into free space. It is, therefore, necessary to ensure the installation and location of the antenna system are appropriate so that it is not damaged. A crucial factor in increasing safety and efficiency is the appropriate positioning of the ELT transmitter with its antenna and control element when interconnected [3,4,5,6].

A fundamental attribute is the correct positioning of the antenna on the aircraft [7]. The appropriate shaping of the antenna characteristic depends on its polar radiation pattern at a frequency of 121.5 MHz for a ground search rescue system, and a spherical radiation pattern at 406 MHz for a satellite search and rescue system. The solution is experimental measurements of the radiation characteristics of the antenna, which can, with a high probability, determine the appropriate position of the antenna [8]. The correct position of the antenna guarantees a quick and accurate determination of the aircraft’s location, which is necessary to ensure the organization of the rescue [9]. Specifying the location of the ELT system antenna on an aircraft is possible only with knowledge of the theory of antennas and their directional characteristics [10,11,12,13]. This is why the final location of the block of this system and its antenna on the aircraft is subject to criticism and safety analyses without verification by experimental measurements [14]. From this point of view, this article offers a relatively simple and fast solution for measuring the polar and spherical characteristics of antennas on small aircraft. Such experimentally validated measurement of antennas in the open space of an airport may be suitable for a broad group of small aircraft manufacturers or manufacturers of small, unmanned aircraft.

## 2. Materials and Methods

The subsections in this section focus on a nonstandard analysis of the spatial directional characteristics of the antenna of an ELT rescue system on a small two-seater aircraft in the open environment of a grassy airport. As such, Section 2.1 describes the area of a suitable layout and the orientation of the measuring setup; Section 2.2, outlines the areas of suitable placement of the electronic devices of the measuring setup. Section 2.3 presents a method for measuring polar directional characteristics at different positional angles in the aerodrome terrain, from which it is possible to determine a spatial directional characteristic. The presented relatively simple and experimentally verified method allows theoretically analyzing and experimentally locating the optimal location for the onboard antenna of an ELT system [12].

### 2.1. Location and Orientation of the Measuring Workplace at the Airport

**Figure 2:**
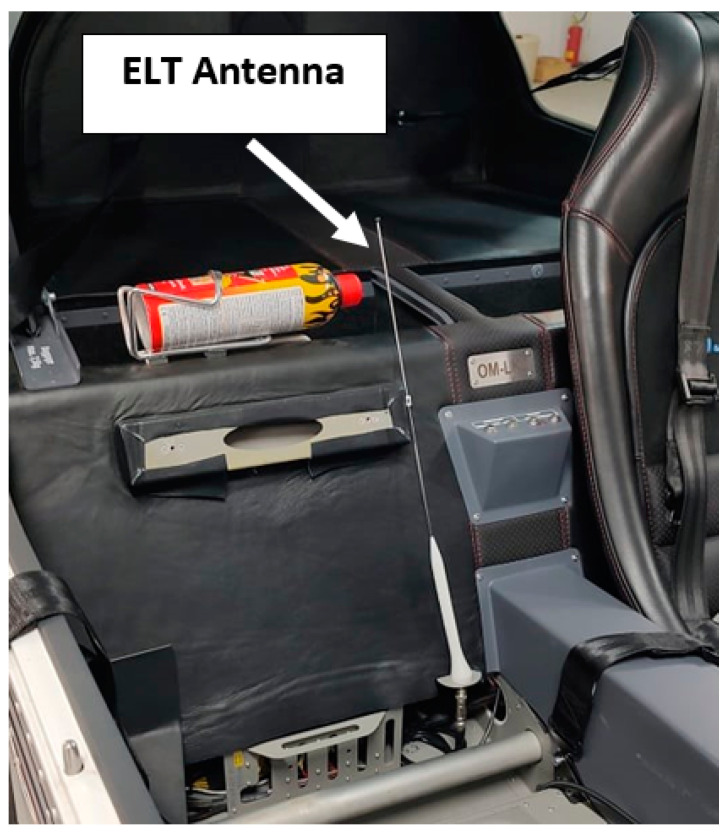
Location of the ELT antenna in a Tomark Aero s.r.o. Viper SD-4 RTC aircraft.

We aimed to evaluate the spherical characteristics of an ELT system antenna after its installation on a small two-seater all-metal aircraft. The contracting authority was the company that manufactures these aircraft. During the structural design of the aircraft, it was optimal to place the antenna in a nonstandard place: in the cockpit space, behind the seat of the right pilot, closer to the central metal panel of the cabin (Figure 2) [15].

This nontraditional placement of the ELT system antenna requires an objective evaluation of its directional radiation characteristics. By placing the antenna in an enclosed space, in the vicinity of which there are metallic and nonmetallic objects and, during the flight, the person piloting the aircraft, it is assumed that the directional characteristic will have a more complex shape. Experimental evaluation of the directional characteristics of the antenna was carried out in an open flat space of a small sports nonpublic airport, Ražňany (LZRY). The locations of the nearest buildings, hangars, and other height obstacles were taken into account when locating the directional measurement measuring station at the airport. The location of the workplace had to provide the possibility of placing the aircraft and manipulating it [12].

**Figure 3:**
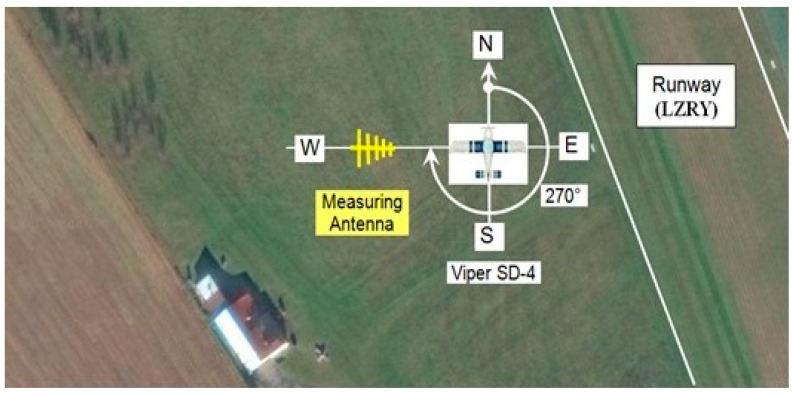
Orientation of the measuring workplace at Ražňany airport.

In the described experiment, the nearest building from the measured aircraft was at a distance of about 130 m. The distance and position of the measured aircraft from the mentioned building at Ražňany Airport are shown in Figure 3. The receiving antenna of the field strength meter was placed at a constant distance of 15 m from the axis of rotation of the aircraft. The aircraft was rotated clockwise so that the evaluated course gradually increased in the range of 0°–360° in steps of 15°. To minimize the effect of the reflected signal from the surrounding buildings, the receiving antenna was rotated by the rear lobe on these buildings, i.e., at an angle of 270° to the axis of the aircraft when it was turned north. Due to the simple and unambiguous reading and writing of the aircraft’s course using the onboard compass in the measurement, the basic direction of the aircraft, in terms of its longitudinal axis, was oriented north at an angle of 0° (Figure 3).

When using an aircraft flight compass to evaluate the course of an aircraft, during the measurement of the antenna’s characteristic onboard the aircraft, it is necessary to emphasize a certain disproportion of angular values. This is a disproportion between the evaluated and the recording measurement angle. If the measurement starts with the orientation of the longitudinal axis of the aircraft to the north, below the course 0° when it is rotated clockwise, the value of the evaluated course of the onboard compass will increase as follows: 0°, 15°, 30°, 45°, etc. At the same time, under the same conditions, the value of the measurement angle will be recorded in the directional characteristic decreasing from the position of the receiving antenna 270° as follows: 270°, 255°, 240°, 225°, etc.

### 2.2. Description of the Location of the Electronic Equipment When Measuring the ELT System

**Figure 4:**
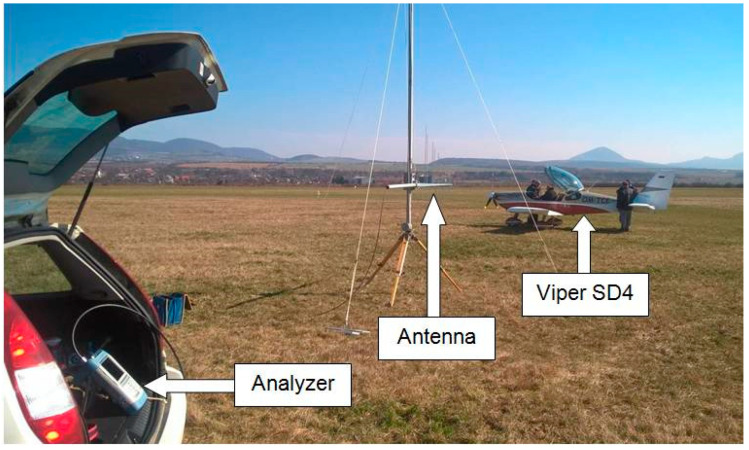
Arrangement of the measuring setup at Ražňany Airport.

The measuring setup created by placing electronic devices around the aircraft at a selected location of the airport is shown in Figure 4. A broadband logarithmic-periodic antenna mounted on a stand was used as the measuring antenna, which could be used to continuously change its height [14,16]. This measuring antenna was placed at a distance of 15 m from the aircraft. A spectrum analyzer placed in a car was used as an electromagnetic field level meter, where the course was recorded and the value of the electromagnetic field was assigned. The vehicle was at a distance of about 8 m from the measuring antenna.

**Figure 5:**
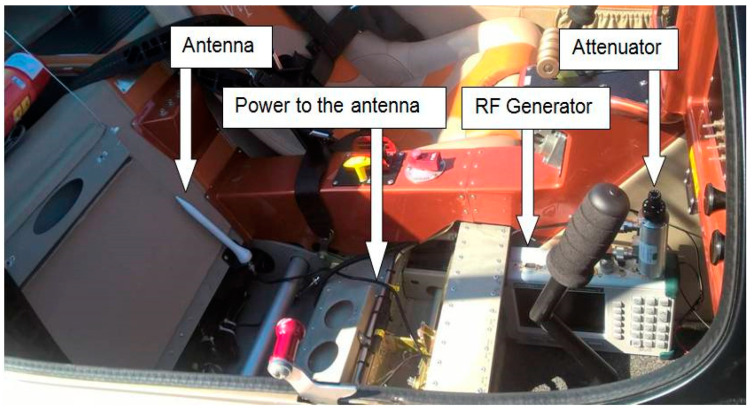
Location of the HF generator in the cabin of the aircraft.

A high-frequency (HF) generator with an attenuator was used as the source of the transmitted signal. The generator was placed in the aircraft cabin and connected to the measured antenna of the ELT system (Figure 5). The aircraft was placed on a simple compensation ring designed to compensate for radio compasses. Due to the design of the Viper SD-4 RTC aircraft [15], measurements were performed in an open environment with a minimum number of obstacles and surrounding buildings. The situational layout of the measured aircraft and the measuring setup at the airport area are illustrated in Figure 5. The measuring frequency was shifted a few megahertz units lower to the frequency not used in the given space and time. Dispatchers at nearby airports were informed about the measurement.

### 2.3. Methodology

We were interested in revealing the radiating properties of the antenna (located in the interior of the cabin), especially at small position angles. At small position angles (as in this case), the metal walls of the cabin shielded the radiation of the antenna to a greater extent. However, the presented method of measuring characteristics, which will be described in the following subsections, can also be used at larger position angles in the range up to 90°. For achieving this, a smaller and lighter measuring antenna (e.g., a five-element Yagi antenna) would have to be used, which has a maximum dimension *D* of approx. 0.6 m. With this antenna size, the far-field *d_F_* value is 1.5 m. Additionally, a simple and still relatively low wooden structure could then be used for measuring the field intensity at large position angles, with the ability to perform measurement even directly above the aircraft.

Figure 4 depicts the layout of the measuring station created for the purpose of directly measuring the spatial radiation characteristics of the antenna on a small aircraft. It is clear from the figure that one part of the electronic device is stationary, and the other is nonstationary. The stationary electronic device is a measuring antenna on a stand and an electromagnetic field strength meter. The nonstationary electronic device is a transmitting (measured) antenna with an HF signal generator located on the measured aircraft. This subsection deals with a relatively simple method of directly measuring the spatial characteristics of the antenna on the aircraft. When measuring it, it is necessary to ensure the rotation of the measured aircraft by changing its azimuth in the horizontal plane, and the mutual change in the position angle between the transmitting and receiving antenna in the vertical plane [12,13].

When objectively measuring the directional characteristics of antennas, it is important to keep a minimum distance between the measured and measuring antenna. This minimum distance, called the beginning of the far field, is defined by:

(1) $$d_{F}\geq \frac{2D^{2}}{\lambda }$$

where *d_F_* is the far field of the antenna, *D* is the maximum dimension of the antenna, and *λ* is the wavelength of the operating frequency.

At the maximum dimension of the broadband logarithmic-periodic measuring antenna (*D* = 1.2 m) and the wavelength *λ* of 0.74 m (at an operating frequency of 406 MHz), the far field starts at a distance of 3.9 m. This minimum distance was respected when measuring in this study.

**Figure 6:**
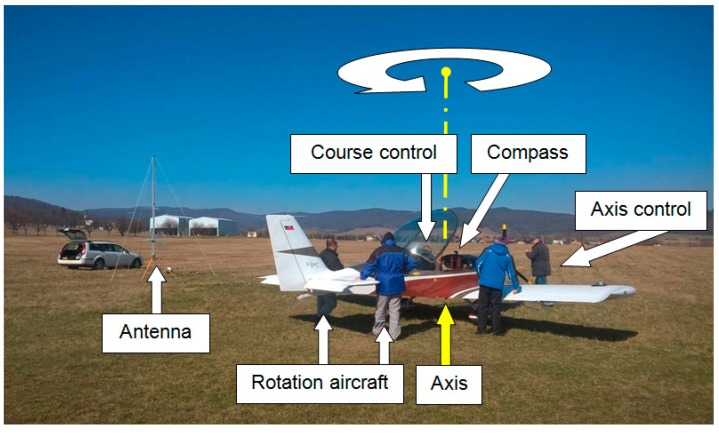
Activities of technical personnel in handling the aircraft during measurements.

The experimental measurements were performed in four series; the position angle between the transmitting and receiving antenna changed for each series of measurements. During the measurement of the series, this angle did not change: it was stationary. In one series of measurements, the azimuth of the aircraft changed with a step of 15°; the azimuth was nonstationary. During all four measurement series, it was necessary to change the position (azimuth) of the aircraft 96 times. Several staff members provided this activity. The onboard compass tracked its course, and the constant position of the axis of rotation was tracked by a plumb line on the aircraft relative to a fixed point on the ground (Figure 6).

The first series of measurements at the low position of the measuring antenna took place in the range of 360° with a step of 15°, and was realized at the lowest position of the receiving (measuring) antenna, placed on a measuring stand at the height of 1.5 m. The height of the heel of the transmitting (measured) antenna of the ELT system was approximately 0.9 m in the aircraft’s cabin. The height difference between the measured and measuring antenna was about 0.6 m. Thus, their distance of 15 m represent only a very small positional angle of measurement of the antenna (*θ* = 2°) of the ELT system in the vertical plane. This created a very shallow directional polar characteristic in the horizontal plane from a spatial point of view.

**Figure 7:**
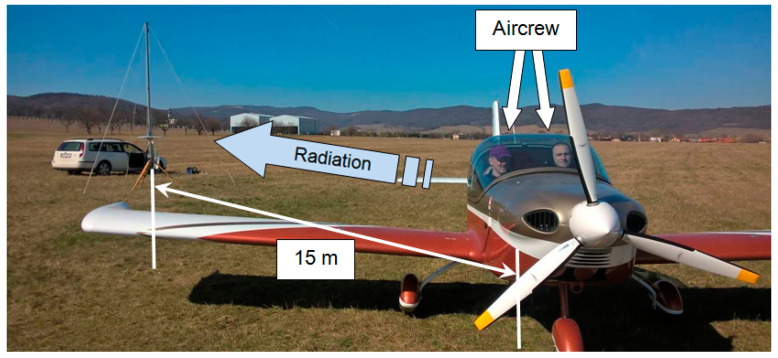
Measurement at a small position angle *θ* of 2° at a distance of 15 m.

The measured characteristics express the radiation of the ELT system antenna from the Viper SD-4 RTC aircraft just above the horizon of the earth’s surface. The actual situation at the specific, i.e., low, position of the receiving antenna on the measuring stand at a distance of 15 m between the aircraft and the stand is shown in Figure 7. Each measurement took place over the entire 360° range and was performed twice for the first time without pilots and for the second time with persons onboard the aircraft.

The second series of measurements at the high position of the measuring antenna also took place in the range of 360° in 15° steps. The measurements were obtained at the uppermost position of the receiving (measuring) antenna, placed on a measuring stand at the height of 5 m. The location of the measured antenna of the ELT system in the cabin of the aircraft was at the same height above the ground, i.e., 0.9 m. The height difference between the measured and measuring antenna was about 4.1 m. Therefore, their distance of 15 m represents a medium position angle of measurement of the antenna (*θ* = 15°) of the ELT system in the vertical plane. From a spatial point of view, a funnel directional polar characteristic was created in the horizontal plane. The measured characteristic expresses the radiation of the ELT system antenna from the Viper SD-4 RTC aircraft at an angle of 15° above the horizon of the Earth’s surface. The measurement was performed using the same scenario as in the previous case. When changing the measurement angle (during the preparation of the next measurement), the operating personnel had to turn a relatively heavy aircraft on slightly bumpy terrain. It was rotated in the form of moving the aircraft back and forth. A fixed positioning system was created to ensure the same position and distance between the transmitting and receiving antenna. A small metal pin was inserted into the ground to ensure axial compatibility, and a small metal weight (plumb) was hung on the aircraft from below under the antenna [17,18].

**Figure 8:**
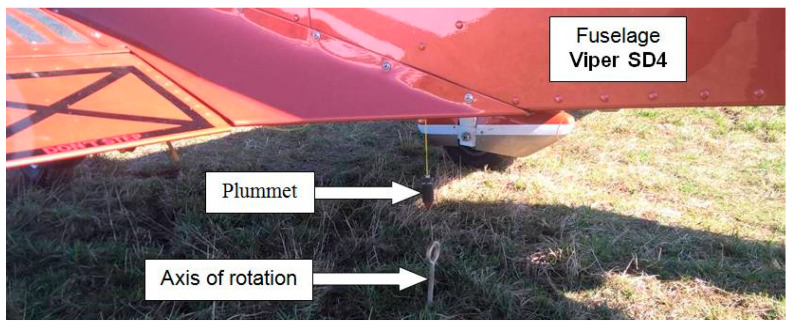
Method of monitoring the axial unambiguity of the aircraft position.

According to the onboard compass, the operators had to turn the aircraft (by 15°) to the new course position and, at the same time, the aircraft had to be placed with an under-fuselage weight above the metal pin (Figure 8).

**Figure 9:**
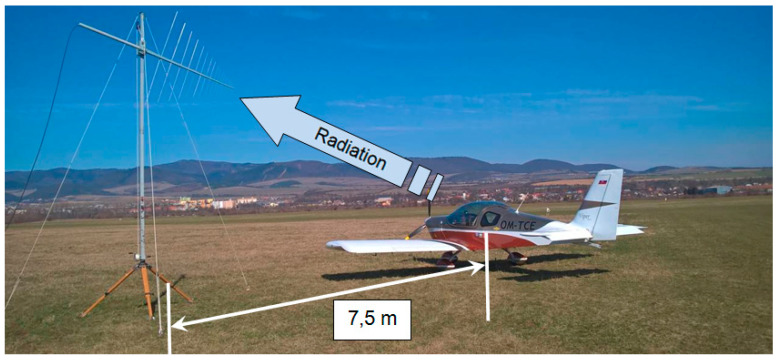
Measurement at a large position angle of *θ* = 30° at a distance of 7.5 m.

The third series of measurements at the high position of the measuring antenna also took place in the range of 360° in 15° steps. The measurement was performed at the uppermost position of the receiving (measuring) antenna, placed on a measuring stand at a height of 5 m. The location of the measured antenna of the ELT system in the cabin of the aircraft was at the same height above the ground, i.e., 0.9 m. The height difference between the measured and measuring antenna was about 4.1 m. However, their half distance of 7.5 m represents a large positional angle of measuring the antenna (*θ* = 30°) of the ELT system in the vertical plane (Figure 9).

This created a deep funnel polar directional characteristic in the horizontal plane with the top of the funnel facing the ground. The measured characteristic expresses the radiation of the ELT system antenna from the aircraft in a high horizon above the Earth’s surface at an angle of 30° toward the sky. At this and larger beam angles, the coverage of the side metal walls of the aircraft cabin is already minimized. For this reason, we wanted to determine the influence of persons onboard on the shape of the antenna’s radiation, which was the aim of the fourth set of measurements. The measurement was again performed according to the same scenario as in the previous cases.

The last (fourth) series of measurements was captured at a high antenna position of 5 m at a distance between the antennas of 7.5 m. Technically and organizationally, the measurement took place in the same way as in the third series of measurements. The only difference was that two people were sitting in the cockpit during the measurements, representing the occupation of the cockpit. A deep funnel polar directional characteristic was also created in the horizontal plane, with the top of the funnel in the measured antenna facing the ground. This measurement demonstrated the influence of persons (human bodies) on the change in the shape of the radiation characteristic at a frequency of 400 MHz [19].

**Figure 10:**
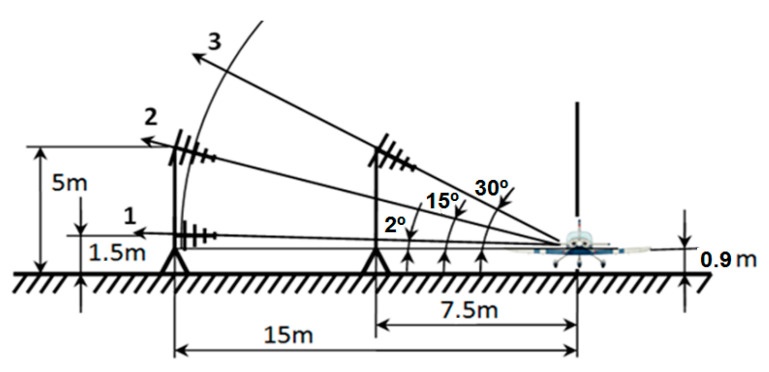
Position measurement angles of *θ* = (2°, 15°, and 30°).

The first three series of measurements were to conducted to obtain an overall spatial idea of the spherical radiation characteristics of the antenna of the ELT rescue system. A spatial summary of all three measurements in terms of antenna distribution and positional angles is shown in Figure 10. From this point of view, the fourth series of measurements was only an additional measurement to illustrate the influence of persons on the shape of the radiation characteristic of the antenna [20].

## 3. Results and Discussion

**Figure 11:**
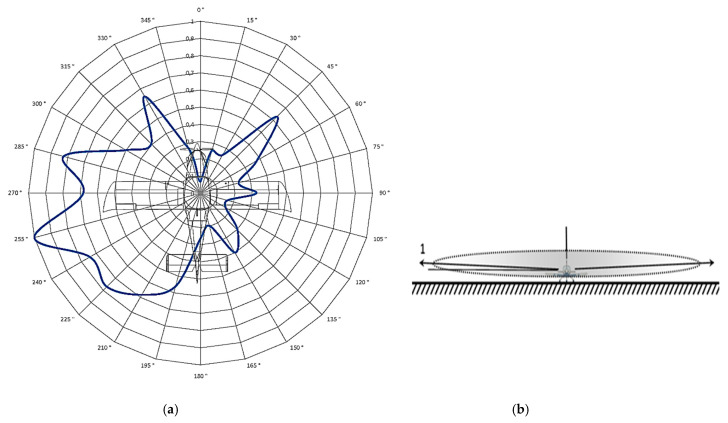
Measurement of the aircraft antenna characteristic at a position angle *θ* of 2°: (**a**) polar characteristics of the antenna; (**b**) spatial illustration of the measurement.

**Figure 12:**
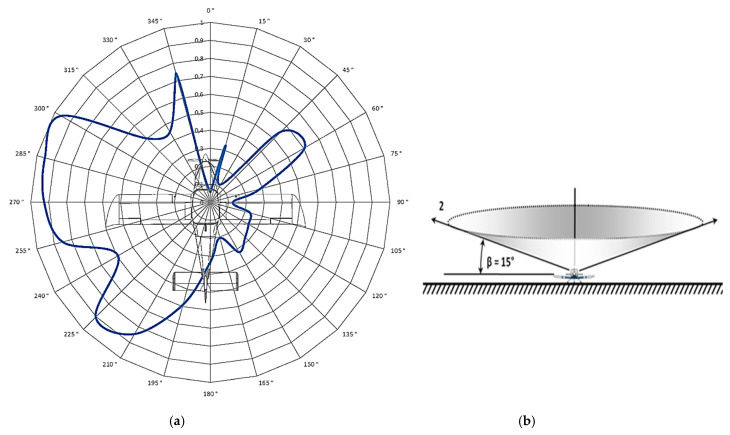
Measurement of the aircraft antenna characteristic at a position angle *θ* of 15°: (**a**) polar characteristics of the antenna; (**b**) spatial illustration of the measurement.

**Figure 13:**
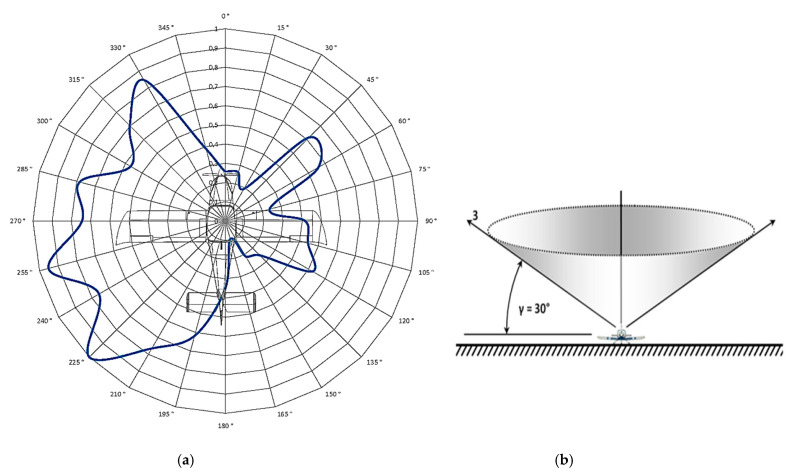
Measurement of the aircraft antenna characteristic at a position angle *θ* of 30°: (**a**) polar characteristics of the antenna; (**b**) spatial illustration of the measurement.

After measuring the directional characteristics of the antenna at different position angles *θ* (2°, 15°, and 30°) with 15° steps in the direction of the aircraft in the range of 0° to 360°, a total of 96 measurements were evaluated. This means that the aircraft was rotated 96 times and its position was finely adjusted by hand. After processing these data, four directional radiation characteristics of the ELT system antenna were created (in polar coordinates). Of the first three characteristics, each one corresponds to a measurement at a different position angle (Figure 11, *θ* = 2°; Figure 12, *θ* = 15°; and Figure 13, *θ* = 30°). The change in the position angle was realized by changing the height and position of the measured antenna with respect to the aircraft. To achieve the required measuring angle, the measuring antenna had to be brought closer to the aircraft.

**Figure 14:**
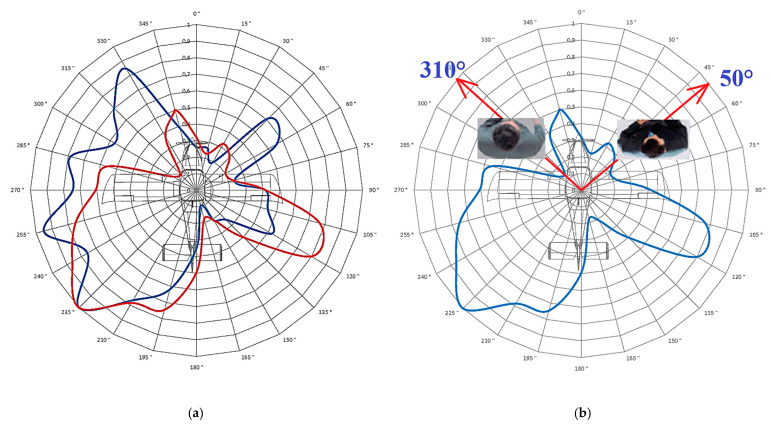
Influence of persons on the characteristic at an angle *θ* of 30° (blue curve indicates without crew, red curve indicates with crew): (**a**) comparison of measured characteristics; (**b**) influence of the position of persons on the shape of the characteristic.

Figure 14b presents the results of the measurement under the same conditions of the spatially placed antenna as in the previous measurement (Figure 13). Thus, measurement was performed at a distance of 7.5 m with an antenna height of 5 m while the antenna was rotated at an angle of 30° downward to the aircraft. During this measurement, two people were seated in the cabin of the aircraft, which created additional shielding of the antenna’s radiation.

**Figure 15:**
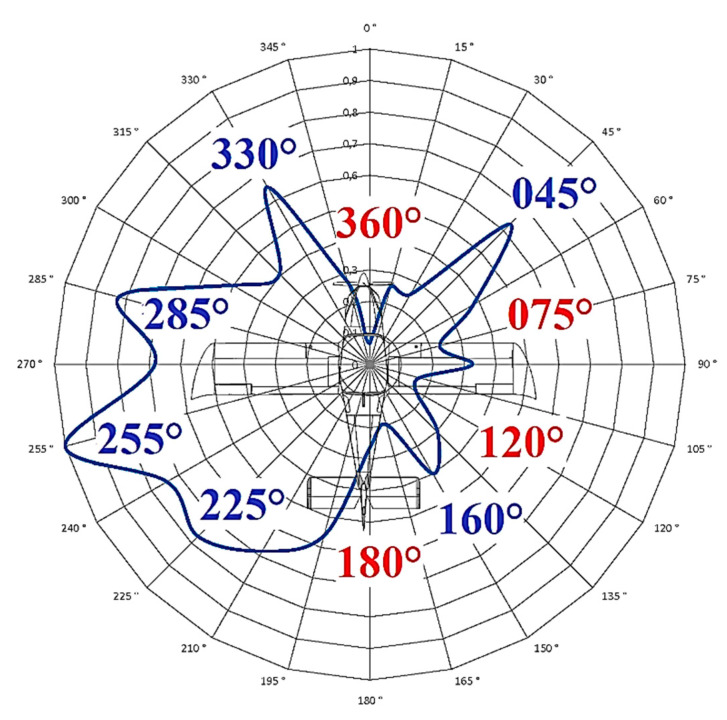
Basic shape of the characteristic at an angle *θ* of 2° with maximum and minimum radiation marked.

Figure 14a shows a comparison of the characteristics in Figure 13 and Figure 15b in the same polar coordinates and with the same spatial setup of the antenna measurement. In the first case (blue) the measurements were recorded without crew and the second case (red) is the measurement with a two-member crew.

Figure 11a, Figure 12a and Figure 13a represent the antenna characteristic, measured at a given position angle. Figure 11b, Figure 12b and Figure 13b depict a spatial illustration presenting the funnel shape of the distribution of the characteristic.

The values using position angles (*θ)* 2°, 15°, and 30° were evaluated mathematically from the defined values of distance, antenna height (cotangent), and antenna height of the ELT system on the aircraft (1 m). After calculation, these values were rounded, and are shown in Table 1.

The assessment of the radiation characteristics of the ELT system antenna located in the aircraft cabin is based on the measured results presented by the four characteristics illustrated in Figure 11, Figure 12, Figure 13 and Figure 14.

There is no exact procedure for the analysis of radiation characteristics. Therefore, only practical experience in the field of antenna theory and antenna measurement backed by theoretical knowledge can be used. The analysis is based on the characteristic in Figure 11, the shape of which has a certain degree of tolerance to each other measured characteristic. For the analysis, the selected section was supplemented with important courses (Figure 15).

The analysis is based on the characteristic in Figure 11, the shape of which can be assigned a certain degree of tolerance to each of the other measured characteristics. For the purposes of the analysis, section of characteristics supplemented with important courses was selected (Figure 15).

None of the real antenna characteristics, even located on the surface of the fuselage, showed an ideal circular shape: a certain whole-circle course of alternating maximum and minimum radiation values was observed. We only assessed the extent to which these maximum and minimum emission values alternate. From this point of view, Figure 15 shows that the measured radiation characteristic can be divided into the right part with a lower level of radiation and the left part with a higher level of radiation. On the left side, there are four distinct radiation maxima, among which there are three indistinct radiation minima. These are maxima on the 225°, 255°, 285°, and 330° courses. On the right side, it is possible to observe three indistinct maxima, between which there are two significant minima of radiation at 75° and 120°. The alternation of the maximum and minimum radiation on the left side of the characteristic is favorable for the position of the antenna, but the radiation on the right side of the characteristic is not favorable for the position of the ELT antenna in the cockpit. It is clear from this figure that the above characteristics have significant minima even at 360° and 180°. These two minima can be also found on radiation characteristics of aircrafts when ELT antenna is mounted classically on the top of the fuselage. In our case, the minimum of the radiation below 0° (360°) was caused by a raised metal dashboard located higher than the top of the antenna.

**Figure 16:**
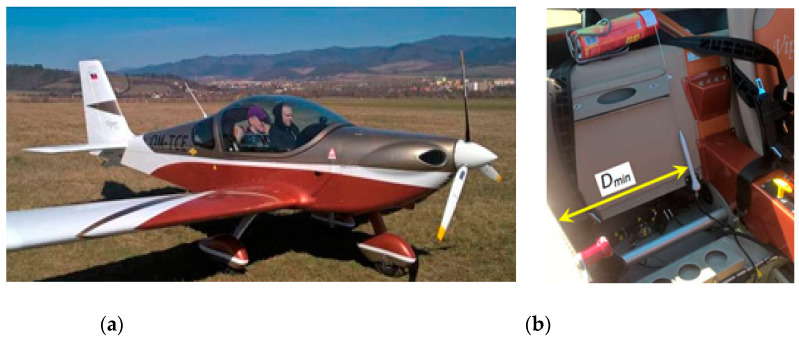
The influence of metal parts of the fuselage on the radiation characteristics; (**a**) the influence of the tail area of aircraft and the rear area of the cockpit; (**b**) the influence of the side wall of the cockpit.

The minimum radiation at 180° is common and can also be seen on different aircraft. It is mostly caused by the shape of the tail of the aircraft. In our case, the raised (metal) rear part of the cockpit, which was also higher with respect to the top of the antenna, contributed to this minimum (Figure 16a).

The lower level of radiated power of the antenna signal directed to the right side of the aircraft is only about one-third of the power directed to the left side of the aircraft. This lower power level on the right side is due to the closer position of the right metal side wall of the aircraft cockpit *D_min_*. This creates a higher level of shielding in this direction, as shown in Figure 16b.

If the effect of the metal wall was negative, this radiation characteristic was measured at a very small position angle (*θ* = 2°). However, at such a low position angle, even after an emergency landing/accident, the 406 MHz Cospas-Sarsat rescue system will not be able to evaluate the aircraft’s position (due to relative position of satellites to the ground). The following radiating directional characteristics of the antenna, measured at a larger position angle, will provide better guidance in this regard.

Figure 11a, Figure 12a and Figure 13a clearly show that, with a certain tolerance of their shape, all three characteristics are approximately the same. Therefore, it can be stated that the same description above applies to all characteristics.

However, it can be observed from these characteristics that the proportion of radiation to the right side of the aircraft also increases with increasing positional angle of the measurement. This is due to the fact that as the position angle increases, more and more free space is exposed for the antenna to radiate from the aircraft cockpit toward the sky.

**Figure 17:**
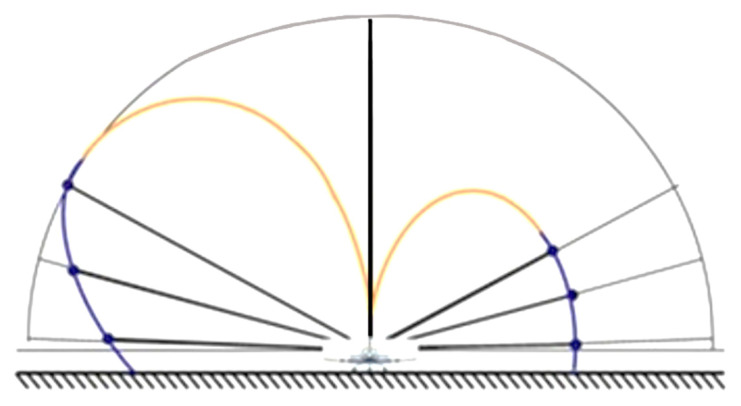
Section of a part of the spherical characteristic of the antenna in the vertical plane.

To confirm this positive statement, a section of the spherical directional characteristic of the antenna radiation in the vertical plane was constructed from the three measured polar directional characteristics. The directional characteristic in the vertical plane was constructed as a section of three radiation characteristics passing through one specified course. For this purpose, a section in one plane was chosen, passing through 45° and 225°. The measured values are displayed in the given angles as vectors having direction and size (Figure 17).

In Figure 17, the actual measured values are marked blue (in the lower part). The estimate of the development of the magnitude of the signal level with increasing position angle is marked in orange (in the upper part). From the obtained diagram, it is clear that on the left side, with increasing position angle, the signal level also significantly increases.

Its maximum value culminates at a position angle in the range of 30° to 45°. On the right side, the signal is shielded, so that it does not reach the level of the signal on the left side. This occurs because, after exceeding the positioning angle of 45°, the signal level decreases even at the ideal radiation characteristic of the vertically polarized antenna, even on those without any shielding.

The fourth measured characteristic (Figure 14b) was obtained from a measurement scenario in which two persons sat in the cockpit (on the seats, directly in front of the antenna system). The presence of persons reduced the level radiation from the antenna (toward the seated persons). The person sitting on the right, i.e., closer to the antenna, created a shield with a minimum signal emission at an angle of 50°.

The decreases in the signal level in the 50° and 310° directions represent the direction from the antenna toward both seated persons. The presence of people greatly reduces the signal level in certain directions defined above. To better understand the influence of the presence of persons on the change in the directional characteristic of the antenna, Figure 14a was created to enable a comparison of characteristics. It is clear from this characteristic radiation that the presence of persons attenuates the signal at defined angles. However, it also increases the signal level toward the back of people, for example, at an angle of 225° on the left side of the characteristic radiation and at an angle of 115° on the right side. 

The measurement of the directional characteristics of antennas in an open field has a number of advantages, but also one serious disadvantage in terms of the reliability of the measured values: the influence of the Earth’s surface on the formation of radiation characteristics of the measured antenna. However, there are several ways to eliminate this negative influence. One way is to place the measuring and measured antenna on a tall mast or tower so that the ground is above the radius of the Fresnel zone of the wireless-enabled link. This principle is used by some companies that professionally deal with the problem of measuring aircraft antennas. When it is not possible to measure large aircraft, we can use scaled-down models that are easier to handle during measurement. This principle could not be used in this study. Instead of we used Snell’s law to eliminate reflections from the ground, in combination with a sunken antenna in the cabin and a relatively small distance between the measuring and measured antennas. The transmitting antenna was positioned so that the radiated wave, after its diffraction on the cabin wall (in the direction where there are no wings or fuselage), did not point to the ground in the area between the measured and measuring antenna. Taking into account Snell’s law, the reflection from the ground did not affect the measurement. When looking at Figure 17, when radiating at an angle of 225° (Figure 15), we can see that in the region between the wing and the fuselage, the level of the measured signal decreases and does not increase as it should with the reflection from the ground. In other directions, the metal surfaces of the wings and fuselage, with a relatively small measuring distance, prevent the application of Snell’s law for signal reflection from the ground.

## 4. Conclusions

In this study, we focused on a specific method of direct experimental measurement of the radiation characteristics of an aircraft antenna on an aircraft to obtain a realistic idea of the shape of its spherical characteristics. At the same time, the aircraft antenna of a small sports aircraft was placed in an unusual place: in its metal cabin. In the described case, it was necessary to evaluate to what level the antenna of the ELT aircraft rescue system radiates toward the satellites of the international search and rescue system Cospas-Sarsat. The presented solution for measuring the antenna characteristics was adopted for several reasons. The main reason is that the small aircraft is so large that it is not possible (in terms of its relatively large dimensions) to place it in a commonly available nonreflective attenuation chamber. Another reason is that only large companies can afford this type of chamber near the airport; for a small manufacturer, this would be an unprofitable investment. For this reason, a scale model of an aircraft is usually used to measure the characteristics of aircraft antennas. However, due to the unusual placement of the antenna in the cabin in connection with the shape of the cabin and the deployment of materially diverse equipment, it would be difficult to achieve credible results.

Methods of practical measurement of directional characteristics of the antennas themselves are well-developed. A problem arises if the antenna measurement must be performed on the object on which the antenna is mounted. An aircraft is a shape-specific means of transport, and its shape significantly changes the shape of the antenna’s radiation characteristics. This is when computer simulations or measurements on scale models of aircraft can be applied, but there may be cases where this a solution may not be optimal. The diversity of the shape, materials, and equipment of the means of transport and, last but not least, the necessary financial security, play a role in this. The benefit of the presented method is that it provides a possible alternative solution to the presented problem. This method can be used by smaller companies, who, given the size of the manufactured object with antennas and the terms of territorial, construction, or financial requirements, cannot invest in suitable equipment. Another benefit is the method of measuring the spatial directional characteristics of the antenna in an airport area, which made it possible to express the spherical radiation characteristic of the measured antenna from several polar characteristics obtained by changing the arrangement of the measuring setup.

The presented spatial radiation characteristics of the antenna were measured in real conditions on a real aircraft. Measurements in the azimuth took place in the range of 0° to 360°, but measurements in the position angle took place in a range only up to 30°. Therefore, the presented measurement method in terms of azimuth range and measurement step has no limitations. However, the method of measuring in the range of the position angle 0° to 90° has a partial technical limitation concerning the complexity of the technical solution of a suitable mounting of the measuring antenna above the aircraft in the position angle range of 45° to 90°. For this reason, the range of antenna measurements on the aircraft at a position angle was limited in this study. However, in the case of additional funds for the project, it is possible to technically solve this problem.

## Figures and Tables

**Table 1:** Calculated values of distances and angles used in measurements.

Position Angle *θ*	Measuring Antenna Height	Distance	Calculated Value	Rounded Value
2°	1.5 m	15 m	1.9°	2°
15°	5 m	15 m	14.9°	15°
30°	5 m	7.5 m	28°	30°

## Data Availability

Data sharing is not applicable.
